# BEATSCORE: Beat-Synchronous Contrastive Alignment and Event-Centric Grading for Long-Term Sports Assessment

**DOI:** 10.3390/s26072157

**Published:** 2026-03-31

**Authors:** Lijie Wang, Jianyong Zhu, Houlei Wang, Xiaochao Li

**Affiliations:** 1 Department of Physical Education, Nanjing University of Posts and Telecommunications, Nanjing 210042, China; 2 School of Cyber Science and Engineering, Nanjing University of Science and Technology, Nanjing 210094, China

**Keywords:** long-term sports assessment, audio–visual synchrony, contrastive learning

## Abstract

Long-term sports assessment is a challenging task in video understanding, since it requires judging subtle movement variations over minutes and evaluating action–music coordination. However, in many sporting events the background music is only weakly related to the performed movements, and the cues that matter for synchrony are often temporal and structural, such as small phase or tempo deviations that occur around decisive moments, rather than semantic correspondences between audio content and action categories. Prior approaches typically rely on implicit cross-modal fusion over dense sequences to learn such weak associations, which can smooth out near-miss misalignment and become brittle under tempo or phase shifts. To address this issue, we propose BEATSCORE, a beat-guided audio–visual learning framework that explicitly models action–music alignment at the beat level and performs event-centric sparse grading for long videos. In our framework, we first convert audio and motion into beat-synchronous tokens, enabling direct comparison on a unified rhythmic timeline. We then introduce a beat-level contrastive objective with near-offset hard negatives to sharpen sensitivity to misalignment. To handle the sparsity of decisive moments, we further design an event proposal and grading module that scores a small set of key segments and aggregates them via learnable multiple-instance pooling into a final assessment score. We evaluate BEATSCORE on public long-term sports benchmarks to demonstrate improved accuracy with competitive efficiency.

## 1. Introduction

**Figure 1:**
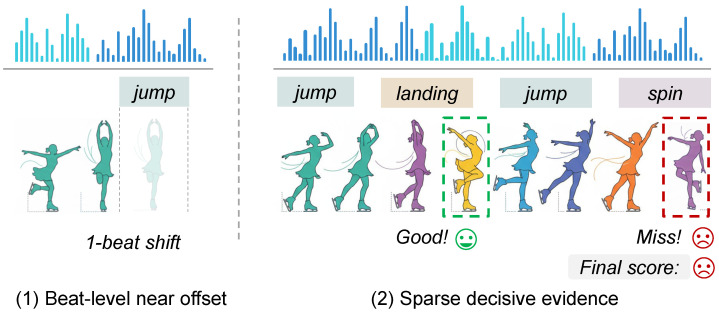
Motivation: near-offset coordination cues and sparse decisive evidence in long-term sports assessment.

Long-term sports assessment aims to predict an overall performance score for minute-long routines, such as figure skating [1,2,3], rhythmic gymnastics [4], or other sports where judging depends on both movement quality and presentation. Unlike short action recognition [5], this task requires comparing subtle differences in technique, stability, and temporal coherence over long durations, while also accounting for action–music coordination that can strongly influence perceived quality and final scores. From a multi-modal sensing perspective, this problem involves the joint interpretation of heterogeneous temporal observations from visual and acoustic streams, where informative cues are often weak, noisy, and unevenly distributed over time. Recent progress has been driven by stronger video backbones [6,7] and multi-modal learning [2,8,9,10,11], highlighting the potential of audio–visual sensing for sports assessment. However, long-term assessment remains challenging because the evidence that determines the score is sparse and often depends on fine-grained temporal structure. In this sense, the task is not only a high-level video understanding problem but also a challenging long-horizon temporal sensing problem that requires robust cross-stream alignment and reliable localization of score-critical moments (Figure 1).

A central difficulty lies in how background music functions in real routines. In many benchmarks, the audio track is not a direct acoustic consequence of the athlete’s movements; instead, it provides a rhythmic scaffold that the choreography follows only loosely [12]. This weak coupling makes the audio stream a complementary sensing signal rather than a direct semantic description of motion content. As a result, the cues that matter for synchrony are not semantic correspondences between audio content and motion categories but, rather, small local phase and tempo deviations—for example, being slightly early, slightly late, or gradually drifting in speed relative to the surrounding beat structure. Importantly, such misalignment is rarely uniform throughout an entire routine. Instead, it often appears as near-miss errors localized in short windows, such as a landing or a peak transition arriving slightly early or late relative to a nearby beat. For a temporal sensing system, these subtle deviations are easy to smooth out when dense sequences are fused implicitly without explicit alignment constraints. This makes action–music coordination difficult to learn from raw audio–visual streams unless the model is explicitly trained to be sensitive to near-offset temporal structure.

Meanwhile, the long-term nature of the task introduces a second, equally important challenge: scoring is event-driven [13,14,15]. A few decisive segments, such as jumps, landings, spins, or highlighted transitions, can dominate the final judgment, while large portions of the video contribute little beyond providing context. Dense sequence modeling with global regression therefore faces an inherent tension: it must process long videos efficiently, yet the supervision signal is concentrated in sparse moments. In practice, dense cross-modal fusion and uniform temporal aggregation can dilute score-critical evidence, leading to suboptimal assessment accuracy for minute-long routines.

Existing audio–visual assessment approaches [2,9] commonly adopt implicit cross-modal fusion (e.g., cross-attention or late fusion) over dense frame- or clip-level sequences to discover associations between motion and music. While effective in some settings, this paradigm has two limitations for long-term sports assessment: First, without explicit beat-level supervision, models can become insensitive to near-offset beat misalignment and remain brittle under local phase or tempo variations (e.g., shifting the audio by one or two beats). Second, dense modeling provides limited leverage for handling sparse decisiveness: much of the model capacity is spent on long-range context, while the few score-critical moments receive diluted attention. Moreover, the community still lacks a systematic and controllable evaluation protocol—such as shift, tempo, and shuffle perturbations—to verify whether a method truly captures synchrony rather than exploiting spurious cues.

To address these challenges, we present BEATSCORE, a beat-guided audio–visual framework that makes action–music coordination explicitly learnable and performs event-centric sparse grading for long videos. Our framework first converts audio and motion cues into beat-synchronous tokens on a unified rhythmic axis, reducing sequence length while standardizing the comparison granularity. We then introduce a beat-level contrastive alignment objective with near-offset hard negatives to sharpen sensitivity to small beat-level misalignment. To capture the sparsity of decisive moments, we further design an event-centric grading module that proposes a small set of key segments without requiring event annotations, scores them using both motion quality and action–music consistency cues, and aggregates segment scores via learnable multiple-instance pooling into the final assessment. Extensive experiments show that BEATSCORE consistently improves assessment accuracy on long-term sports benchmarks. Our contributions are three-fold:

- We unify audio and motion cues on a beat-aligned timeline via beat-synchronous tokenization, providing a standardized granularity for long-term assessment.
- We introduce a beat-level contrastive objective with near-offset hard negatives to learn fine-grained action–music coordination cues for accurate scoring.
- We design an event-centric grading pipeline that selects and scores a small set of key segments, and then aggregates segment scores via learnable multiple-instance pooling to improve long-term sports assessment accuracy.

## 2. Related Work

### 2.1. Sports Assessment

Sports assessment is commonly studied under the umbrella of action quality assessment (AQA), where the goal is to predict a continuous score reflecting performance quality rather than action categories. Early AQA methods [16,17] typically regress scores from global video descriptors or short clips, but they often struggle to capture subtle quality differences that are temporally localized. To address this, a large body of work emphasizes temporal modeling and aggregation, such as learning quality-aware temporal pooling, exploiting long-range dependencies with recurrent models or Transformers, and explicitly modeling temporal evolution to better represent technique and stability [13,18,19,20,21]. Other approaches decompose a performance into informative parts by segmenting sub-actions, detecting key phases, or using sparse proposals, aiming to avoid treating all frames equally when the supervision is only available at the video level [13,18,19].

Long-term sports assessment further amplifies these challenges because routines are minute-long and typically contain only a small number of score-critical moments (e.g., take-off, landing, peak transition), while the remaining content provides context and continuity [1,14,22]. This has motivated multi-scale and long-range modeling strategies that can integrate information across extended time spans without collapsing everything into a single uniform average [4,14]. At the same time, dense modeling alone does not fully resolve the mismatch between sparse decisive evidence and video-level supervision, which has led to multiple-instance learning and attention-based aggregation for scoring, where the model learns to emphasize a subset of informative clips or segments [23]. Our work follows this line but makes the sparsity assumption explicit at the level of short, rhythm-aligned events, so that segment selection and grading are aligned with how decisive moments affect the final score.

Recent studies also explore multi-modal signals for sports assessment, including audio and language, to inject complementary cues and better align predictions with judging criteria [2,3,9,24,25,26,27]. However, in many long-term sports benchmarks the audio track is background music selected externally, rather than action-generated sound, so useful audio information often appears as rhythmic structure and coordination patterns instead of semantic correspondence between audio content and action categories [1,2,4,28,29]. This motivates approaches that learn fine-grained coordination cues tied to timing and connect them to scoring through event-centric grading rather than uniform dense averaging. In this work, we focus on beat-synchronous modeling and near-offset coordination signals as a principled way to support accurate long-term assessment.

### 2.2. Multi-Modal Video Understanding

Multi-modal video understanding aims to leverage multiple modalities, most commonly vision and audio, to model complex video content [6,27,28,30]. A common design is cross-modal fusion, where audio and visual streams are combined by late fusion, cross-attention, or shared latent representations, enabling each modality to complement the other under noise or ambiguity [29,31,32,33]. Contrastive learning has also become a standard tool for aligning audio and visual representations by bringing matched pairs closer and pushing mismatched pairs apart, improving transfer to downstream tasks [21,34,35,36]. Beyond generic alignment, a related direction studies audio–visual correspondence and synchrony, focusing on temporal consistency and learning signals that distinguish aligned from misaligned streams [36,37]. These methods provide a useful foundation for our setting, where the key coordination cues are temporal and fine-grained.

Language-conditioned multi-modal learning further extends this paradigm by using text as an additional modality or as supervision to shape representations [38,39,40]. Language guidance is particularly effective when textual descriptions capture high-level semantics that are difficult to infer from audio–visual observations alone [41]. However, long-term sports assessment has a different emphasis: background music does not necessarily convey semantic information about the performed actions, and the relevant relation between audio and motion is often expressed through timing and rhythm. Therefore, directly relying on semantic alignment may be insufficient to capture the score-critical coordination patterns. Our approach instead focuses on rhythm-aware modeling by mapping modalities onto a beat axis and explicitly learning near-offset misalignment signals, which are better matched to the nature of action–music coordination in sports routines.

Finally, our method is also related to event-centric modeling for long videos, where only a few segments dominate the final prediction and the model must identify and exploit these segments under weak supervision [23,42,43]. Such approaches typically use proposal mechanisms, saliency scoring, and learnable aggregation to mitigate the dilution effect of uniform temporal pooling. We adopt a similar high-level principle, but we instantiate it on a beat-synchronous timeline and couple it with beat-level alignment learning, so that event selection and grading are grounded in coordination-sensitive representations.

## 3. Method

### 3.1. Overview

We propose **BEATSCORE**, a beat-guided audio–visual framework for long-term sports assessment. Given a routine video *V* and its audio track *A*, we predict an overall assessment score $\hat{s}\in R$. Let $V={\left\{v_{t}\right\}}_{t=1}^{T}$ denote the visual stream sampled into *T* frames with timestamps ${\left\{\tau_{t}\right\}}_{t=1}^{T}$, and let s∈R be the ground-truth score provided at the video level. Let *A* denote the raw waveform of the audio track. BEATSCORE is motivated by two properties of this task: First, action–music coordination is mainly reflected by fine-grained rhythmic structure, where informative differences often appear as near-offset misalignment around key moments. Second, while a few decisive segments can heavily influence the final judgment, long routines also contain distributed evidence such as overall smoothness and consistency, so modeling should capture both event-level cues and holistic quality.

**Figure 2:**
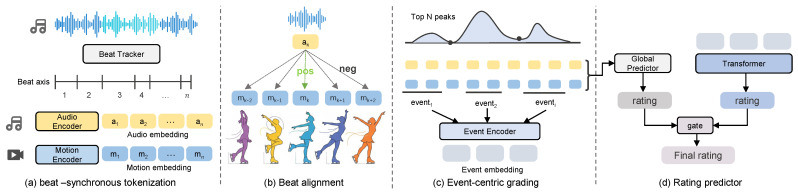
Overview of BeatScore. BEATTOK maps the routine video and its accompanying music track onto a shared beat axis. Beat-level contrastive alignment learns synchronized audio–motion representations with near-offset hard negatives. Event-centric sparse grading aggregates salient local-window scores via MIL, and a global branch with adaptive gated fusion outputs the final score $\hat{s}$.

Our framework has three stages. We first map audio and movement cues onto a unified beat axis and construct beat-synchronous tokens for standardized cross-modal comparison. We then introduce a beat-level contrastive alignment objective with near-offset hard negatives to explicitly learn coordination cues that benefit scoring. Finally, we perform event-centric sparse grading by proposing a small set of key segments on the beat axis, predicting their scores with a lightweight event encoder, and aggregating them via learnable multiple-instance pooling. In parallel, we add a lightweight global grading branch on the same beat tokens and fuse it with the event-centric prediction, so the final score reflects both decisive events and routine-level coherence (Figure 2).

### 3.2. Beat-Synchronous Tokenization

We first unify the temporal granularity of audio and motion by tokenizing both streams on a beat axis. Importantly, we align the routine video with its accompanying music track on a beat axis extracted from the music, rather than aligning action-generated sounds with motion. Using an off-the-shelf beat tracker on *A*, we obtain beat boundaries $B={\left\{b_{k}\right\}}_{k=1}^{K+1}$, where $b_{k}$ is the timestamp of the *k*-th beat boundary and $\left[b_{k},b_{k+1}\right)$ is the *k*-th beat interval. This converts a minute-long routine into a sequence of *K* beat units, typically with K≪T. All subsequent audio–motion comparison and event proposal are performed on this beat axis.

For audio, we compute a fixed-dimensional token per beat interval. We represent each segment $A_{\left[b_{k},b_{k+1}\right)}$ as a log-mel spectrogram (with a fixed mel dimension), feed it into an audio encoder $f_{a}\left(\cdot \right)$ (a lightweight Transformer), and apply temporal pooling on the segment-level features to obtain one token.

(1) $$a_{k}=f_{a}\;\left(A_{\left[b_{k},b_{k+1}\right)}\right)\in R^{d_{a}},\;k=1,\ldots ,K.$$

when beat intervals have different durations, the pooling step ensures that $a_{k}$ has a consistent dimension $d_{a}$.

For motion, we extract frame-level movement features $x_{t}$ from each $v_{t}$ and encode them with a motion encoder $f_{m}\left(\cdot \right)$ to obtain $u_{t}=f_{m}\left(x_{t}\right)\in R^{d_{m}}$. For each beat interval $\left[b_{k},b_{k+1}\right)$, we collect indices $I_{k}=\left\{t|b_{k}\leq \tau_{t}<b_{k+1}\right\}$ and aggregate ${\left\{u_{t}\right\}}_{t\in I_{k}}$ into a beat token $m_{k}\in R^{d_{m}}$ using attention pooling, $m_{k}=\sum_{t\in I_{k}}\alpha_{t}u_{t}$ with $\alpha_{t}={\mathrm{softmax}}_{t\in I_{k}}\left(w^{⊤}\tanh\left(Wu_{t}\right)\right)$, where $W\in R^{h\times d_{m}}$ and $w\in R^{h}$ are learnable. If $I_{k}$ is empty (rare when *T* is large), we set $m_{k}$ to the nearest available token (nearest-neighbor fill) to keep a complete beat sequence. This step reduces the sequence length from *T* to *K* and standardizes the granularity for audio–motion coordination.

### 3.3. Beat-Level Contrastive Alignment

Beat-synchronous tokens provide a unified timeline, but accurate assessment requires the representation to be sensitive to near-offset misalignment at the beat level. We therefore introduce a contrastive alignment objective that treats aligned beat pairs as positives and temporally adjacent beat pairs as hard negatives. We first project audio and motion tokens into a shared embedding space using lightweight heads $g_{a}\left(\cdot \right)$ and $g_{m}\left(\cdot \right)$, ${\tilde{a}}_{k}=\mathrm{norm}\left(g_{a}\left(a_{k}\right)\right)$ and ${\tilde{m}}_{k}=\mathrm{norm}\left(g_{m}\left(m_{k}\right)\right)$, where norm(·) denotes $\ell_{2}$ normalization and ${\tilde{a}}_{k},{\tilde{m}}_{k}\in R^{d}$. We use cosine similarity $\mathrm{sim}\left(p,q\right)=p^{⊤}q$.

For each beat index *k*, $\left({\tilde{m}}_{k},{\tilde{a}}_{k}\right)$ is a positive pair. We construct near-offset hard negatives by pairing ${\tilde{m}}_{k}$ with ${\tilde{a}}_{k+\delta }$, where δ is a small temporal offset. Let D be a fixed offset set (for example, D={−2,−1,+1,+2}); we ignore invalid indices outside [1,K]. We optimize an InfoNCE-style objective with temperature τ:

(2) $$L_{\mathrm{align}}=-\frac{1}{K}\sum_{k=1}^{K}\log\frac{\exp\;\left(\mathrm{sim}({\tilde{m}}_{k},{\tilde{a}}_{k})/\tau \right)}{\exp\;\left(\mathrm{sim}({\tilde{m}}_{k},{\tilde{a}}_{k})/\tau \right)+\sum_{\delta \in D}\exp\;\left(\mathrm{sim}({\tilde{m}}_{k},{\tilde{a}}_{k+\delta })/\tau \right)}.$$

We include in-batch negatives by adding ${\tilde{a}}_{k^{\prime }}$ from other samples in the batch to the denominator, which strengthens the contrastive signal while keeping the positive definition unchanged. This objective provides direct supervision for distinguishing aligned coordination from near-offset misalignment at the beat level.

### 3.4. Event-Centric Grading and Learnable Aggregation

Long-term routines are often influenced by a small number of decisive moments, so we adopt an event-centric grading strategy on the beat axis. We first propose *N* candidate events from motion tokens ${\left\{m_{k}\right\}}_{k=1}^{K}$ using a beat-level saliency signal. We compute saliency by temporal change magnitude $E_{k}={∥m_{k}-m_{k-1}∥}_{2}$ for k=2,…,K (and set $E_{1}=0$). We select event centers ${\left\{c_{i}\right\}}_{i=1}^{N}$ via greedy peak picking with non-maximum suppression on the beat axis: at step *i*, we choose $c_{i}=\arg\max_{k}E_{k}$, and then suppress a neighborhood $\left[c_{i}-\rho ,c_{i}+\rho \right]$ by setting $E_{k}=0$ for *k* in that range, where ρ is a suppression radius in beats. In this work, an event denotes a short beat-aligned temporal window centered at a salient peak on the motion-driven event saliency curve, rather than a manually annotated semantic action unit. Given a window radius *L* beats, the *i*-th event corresponds to

(3) $$e_{i}=\left[\ell_{i},r_{i}\right],\;\ell_{i}=\max\left(1,c_{i}-L\right),\;\;r_{i}=\min\left(K,c_{i}+L\right).$$

This proposal is annotation-free and yields short beat windows of at most 2L+1 in length, making subsequent grading efficient and focused.

For each event $e_{i}$, we gather beat-synchronous tokens ${\left\{a_{k},m_{k}\right\}}_{k=\ell_{i}}^{r_{i}}$ and encode them into an event representation. We form fused beat tokens $y_{k}=\phi \left(\left[m_{k};a_{k}\right]\right)\in R^{d}$ by concatenation and a linear projection ϕ(·), and we add a learnable event token $y_{\mathrm{cls}}\in R^{d}$ to summarize the segment. We also add standard positional embeddings within the event window (indexed by beat offset) to preserve local order. We instantiate the event encoder Enc(·) as a shallow Transformer encoder with $L_{e}$ layers and *H* attention heads, applied to the sequence $\left\{y_{\mathrm{cls}},y_{\ell_{i}},\ldots ,y_{r_{i}}\right\}$. Let $z_{i}\in R^{d}$ be the output embedding at the event token position, and predict an event score with a regressor h(·):

(4) $$z_{i}=\mathrm{Enc}\;{\left(\{y_{\mathrm{cls}},y_{\ell_{i}},\ldots ,y_{r_{i}}\}\right)}_{\mathrm{cls}},\;s_{i}=h\left(z_{i}\right)\in R.$$

Because events are short beat windows and the beat-level contrastive objective already encourages coordination-sensitive features, a shallow encoder is sufficient for segment grading.

We aggregate event scores into an event-centric prediction via learnable multiple-instance pooling. We compute an importance weight $\pi_{i}$ for each event from its representation $z_{i}$ and normalize across the *N* events:

(5) $$\pi_{i}=\frac{\exp\;\left(q^{⊤}\tanh\left(Wz_{i}\right)\right)}{\sum_{j=1}^{N}\exp\;\left(q^{⊤}\tanh\left(Wz_{j}\right)\right)},\;{\hat{s}}_{\mathrm{event}}=\sum_{i=1}^{N}\pi_{i}s_{i},$$

where $W\in R^{h^{\prime }\times d}$ and $q\in R^{h^{\prime }}$ are learnable parameters.

Event-centric evidence alone can miss distributed quality signals that are not concentrated in a few peaks. To capture routine-level coherence, we add a lightweight global grading branch on the full beat sequence. We reuse the fused beat tokens $y_{k}=\phi \left(\left[m_{k};a_{k}\right]\right)$ for k=1,…,K, prepend a learnable global token $y_{\mathrm{cls}}^{g}\in R^{d}$, and apply a shallow global encoder ${\mathrm{Enc}}_{g}\left(\cdot \right)$ (with $L_{g}$ layers, typically $L_{g}\leq 2$) to obtain a global representation $z_{g}$:

(6) $$z_{g}={\mathrm{Enc}}_{g}\;{\left(\{y_{\mathrm{cls}}^{g},y_{1},\ldots ,y_{K}\}\right)}_{\mathrm{cls}},\;{\hat{s}}_{\mathrm{global}}=h_{g}\left(z_{g}\right)\in R,$$

where $h_{g}\left(\cdot \right)$ is a linear regressor. We then fuse the event-centric and global predictions with an adaptive gate:

(7) $$\alpha =\sigma \left(w^{⊤}z_{g}+b\right),\;\hat{s}=\alpha \;{\hat{s}}_{\mathrm{event}}+\left(1-\alpha \right)\;{\hat{s}}_{\mathrm{global}},$$

where σ(·) is the sigmoid function, and $w\in R^{d}$ and b∈R are learnable. This fusion retains the benefits of sparse event grading while allowing the model to account for holistic consistency when it is informative.

### 3.5. Training Objective

We train BEATSCORE end-to-end with a score regression loss and the beat-level contrastive alignment loss. Given the ground-truth score *s*, we supervise the fused prediction $\hat{s}$ with $L_{\mathrm{score}}=\ell \left(\hat{s},s\right)$, where ℓ(·) is $L_{1}$ or Huber depending on the benchmark. The overall objective is

(8) $$L=L_{\mathrm{score}}+\lambda \;L_{\mathrm{align}},$$

where λ balances assessment supervision and beat-level alignment learning, and $L_{\mathrm{align}}$ is defined in Equation (2).

## 4. Experiments

We evaluated BEATSCORE on three public long-term sports assessment benchmarks—FS1000 [2], Fis-V [1], and Rhythmic Gymnastics (RG) [4]—following the standard splits, score normalization, and reporting protocols used in prior work. We report Spearman correlation (SRCC) and mean squared error (MSE). In all comparison tables, we summarize each method’s input modality and backbone in the Features column.

### 4.1. Datasets

We evaluated BEATSCORE on three public long-term sports assessment benchmarks with synchronized routine videos and background music, namely, FS1000 [2], Fis-V [1], and Rhythmic Gymnastics (RG) [4]. All three datasets provide video-level judge scores, and we followed the official splits and evaluation protocols used in prior works for fair comparison.

**FS1000:** FS1000 contains 1000 training videos and 247 validation videos across eight figure skating competition categories. Each routine is recorded at 25 fps and has roughly 5000 frames. In addition to the total scores TES and PCS, FS1000 additionally provides five component scores, namely, Skating Skills (SS), Transitions (TR), Performance (PE), Composition (CO), and Interpretation of Music (IN). Following the standard FS1000 protocol, we treated each score type as an independent prediction target and trained a separate model for TES, PCS, SS, TR, PE, CO, and IN.
**Fis-V:** Fis-V consists of 500 figure skating ladies singles short-program videos. Each video is about 2.9 min long at 25 fps. We adopted the official split, with 400 training videos and 100 testing videos. Each sample was annotated with TES and PCS, and we trained two separate models to predict these two targets.
**RG:** The RG dataset includes 1000 rhythmic gymnastics videos spanning four apparatus types: ball, clubs, hoop, and ribbon. Each routine is about 1.6 min long at 25 fps. The benchmark was organized per apparatus, with 200 training videos and 50 evaluation videos for each apparatus type. We trained one model per apparatus, reporting both per-type and averaged results.

### 4.2. Evaluation Metrics

Following common practice in long-term sports assessment [2,3,12,15], we report Spearman’s rank correlation coefficient and mean squared error (MSE) on the test split for each dataset. Given *N* test videos, ground-truth scores ${\left\{s_{i}\right\}}_{i=1}^{N}$, and predictions ${\left\{{\hat{s}}_{i}\right\}}_{i=1}^{N}$, Spearman correlation measures rank consistency between predictions and labels:

(9) $$\rho \;=\;\mathrm{Corr}\left(\mathrm{rank}\left(s\right),\;\mathrm{rank}\left(\hat{s}\right)\right),$$

where higher is better. MSE measures the numerical deviation in the score space:

(10) $$\mathrm{MSE}\;=\;\frac{1}{N}\sum_{i=1}^{N}{\left({\hat{s}}_{i}-s_{i}\right)}^{2},$$

where lower is better.

### 4.3. Implementation Details

All experiments were conducted on four RTX 4090 GPUs using PyTorch. For fair comparison with prior works, we followed the benchmark-standard feature settings: on **Fis-V** and **RG**, we extracted visual features with a Video Swin Transformer (VST) pre-trained on Kinetics-600 and audio features with an Audio Spectrogram Transformer (AST) pre-trained on AudioSet; on **FS1000**, we used the TimeSformer (TF) and AST features released in prior work to match its established protocol. We normalized the scores to [0,1] per target by $s^{\prime }=s/\xi$, where ξ is the maximum training-set score, and rescaled the predictions back when reporting MSE. Beat timestamps were obtained with the same off-the-shelf beat tracker for all datasets, and both modalities were tokenized on the beat axis. For beat-level contrastive alignment, we constructed near-offset hard negatives by shifting along the beat index with an offset set D={−2,−1,+1,+2} ($\Delta_{\max}=2$). Event-centric grading selects *N* salient peaks via motion-saliency peak picking with NMS (radius ρ in beats) and forms local windows of radius *L* beats; unless otherwise specified, we used N=4, L=3, and ρ=2. Each event window was encoded by a shallow Transformer event encoder and aggregated by MIL pooling to produce the routine-level score. We optimized the objective using AdamW with weight decay $1\times 10^{-4}$ and a cosine learning-rate schedule (final ratio 0.01); the batch size was 64 and the learning rate was $3\times 10^{-4}/3\times 10^{-4}/1\times 10^{-4}$ for FS1000/Fis-V/RG, respectively. To mitigate overfitting, we used dropout 0.3/0.7/0.3 on FS1000/Fis-V/RG, respectively, and trained for different epochs per benchmark following common practice, selecting the best checkpoint on the validation split.

### 4.4. Comparison with State of the Art

**FS1000:** Table 1 compares BEATSCORE with representative visual-only approaches and recent audio–visual models on FS1000 across seven judge scores (TES, PCS, SS, TR, PE, CO, IN). Two high-level observations emerge: First, strong visual encoders already provide a competitive foundation for long-term assessment: Transformer-based visual-only methods (e.g., TF-based baselines) substantially outperform earlier C3D-style pipelines on both SRCC and MSE, especially on TES and PCS. Second, incorporating audio is helpful but not automatically decisive. Compared with visual-only Transformers, naive late fusion may improve some components yet remain inconsistent across score types, suggesting that “audio-action” cues in real routines are weak and highly structured, and are not easily captured by dense cross-modal fusion.

BEATSCORE improves over prior audio–visual methods under the same TF+AST settings, achieving stronger rank consistency (Avg. SRCC) while reducing numerical error (Avg. MSE). Beyond the overall average, the gains are typically more pronounced on components that are sensitive to choreography and music coordination, such as Interpretation (IN), Composition (CO), and Transitions (TR), where temporal structure and near-offset deviations matter more than coarse action semantics. This aligns with our design choices: beat-synchronous tokenization reduces ambiguity about the comparison granularity, beat-level contrastive alignment explicitly trains sensitivity to near-beat mismatches, and event-centric grading focuses learning on decisive segments rather than uniformly aggregating long sequences.

**Fis-V:** Table 2 reports results on Fis-V, which predicts TES and PCS on short programs and provides a complementary setting to FS1000. Fis-V has fewer videos and typically exhibits higher variance across athletes and music tracks, which makes robust learning of audio–motion cues challenging. Despite this, BEATSCORE achieves improved SRCC and reduced MSE over prior methods, indicating that the proposed beat-guided alignment does not rely on extremely long sequences to be effective. We also find that the relative improvement is often larger for PCS than TES. This is expected, since PCS emphasizes components like performance interpretation and composition, which are more likely to be affected by action–music coordination patterns than purely technical execution.

From a practical perspective, Table 2 also reports complexity (Params/FLOPs). BEATSCORE attains better accuracy with a small overhead compared with the underlying TF + AST feature setting. This is important for long-term assessment, where the computational budget is dominated by processing long sequences; our design improves prediction quality without requiring substantially larger backbones or multi-stage refinement.

**RG:** Table 3 summarizes performance on RG across four apparatus types. RG exhibits substantial diversity in movement patterns and accompanying music, and judging is often dominated by a handful of highlighted transitions and peak moments. This makes it a good testbed for our event-centric formulation. BEATSCORE improves the averaged SRCC and MSE across apparatus, with gains typically consistent across ball, clubs, hoop, and ribbon. We additionally observe that visual-only models can be competitive on some apparatus, yet audio–visual methods that explicitly model coordination can yield higher average performance, which supports our view that alignment cues exist but must be extracted at the right temporal granularity.

### 4.5. Ablation on Core Components

Table 8 reports a compact ablation of BEATSCORE on FS1000 and RG under the same feature settings. Each row removes one core component from the full model, allowing us to quantify its contribution while keeping the rest of the pipeline unchanged.

**Effect of Beat-Level Alignment:** Removing the beat-level contrastive alignment loss ($L_{\mathrm{align}}$) leads to a clear drop in SRCC and a corresponding increase in MSE. This indicates that simply fusing audio and motion on beat-synchronous tokens is not sufficient: the model benefits from an explicit objective that discriminates the aligned pair $\left(m_{k},a_{k}\right)$ from near-offset candidates around neighboring beats. In practice, this encourages the representation to be sensitive to small timing deviations that are visually subtle but score-relevant, which then propagates to the final grading head.

**Effect of Event Proposal:** Replacing event-centric proposal with *uniform-N windows* also degrades performance. Importantly, this variant still computes the same number of window-level predictions and uses the same MIL pooling, but it loses the ability to prioritize salient moments. The drop suggests that the gain of BEATSCORE is not merely from reducing the temporal length but from selecting informative windows whose local evidence better correlates with the routine-level score.

**Effect of MIL Pooling:** Finally, replacing MIL pooling with mean pooling causes a noticeable performance decrease. This is consistent with the scoring mechanism in long routines, where different events contribute unequally to the final assessment. A learnable pooling module can assign higher weights to decisive windows and downweight less informative segments, whereas uniform averaging tends to dilute these key signals and yields less accurate predictions.

Overall, the three ablations show that $L_{\mathrm{align}}$, event proposal, and MIL pooling are complementary: alignment improves the quality of audio–motion comparisons, proposal improves the relevance of the evidence being graded, and MIL improves how this sparse evidence is aggregated into a single score.

**Impact of Different Beat Trackers on BEATSCORE:** As shown in Table 4, BEATSCORE remains consistently effective across different beat trackers. While the default tracker, librosa, achieves the best overall performance, Essentia yields highly comparable results on both FS1000 and RG, and aubio also maintains competitive performance, with only a moderate drop. These results suggest that our method does not rely on a specific beat detection tool, although the quality of beat boundary estimation still affects the final grading accuracy.

**Effects of Different Event Proposal Strategies:** Table 5 compares different event proposal strategies. Audio-only proposal consistently underperforms motion-based variants, indicating that rhythm cues alone are insufficient for identifying decisive scoring moments. In contrast, incorporating audio cues into motion-based proposal yields competitive, but not consistently better, performance. This suggests that audio rhythm is best used as a complementary signal rather than as a standalone event selector.

**Effect of Decomposing Action Quality and Action–Music Coordination Signals in Event-Centric Grading:** We further analyzed whether the gains of BEATSCORE mainly come from action quality cues or from action–music coordination. To this end, we compared three variants: (1) quality-only scoring, which uses only motion/visual information; (2) coordination-only scoring, which relies on audio–motion interaction cues; and (3) the full model that combines both quality and coordination signals. All other settings were kept unchanged.

As shown in Table 6, quality-only scoring already provides a strong baseline, while coordination-only scoring performs worse, indicating that motion quality remains the primary cue for long-term sports assessment. However, combining quality and coordination yields the best overall performance on both datasets, showing that action–music coordination provides complementary evidence beyond pure motion quality.

**Robustness of BEATSCORE under Noisy Audio Conditions:** We further evaluated the robustness of BEATSCORE under noisy audio conditions. To simulate realistic deployment scenarios, we injected mixed audio noise before beat extraction and audio feature encoding, such that both beat-synchronous segmentation and downstream grading were affected. We compared three settings: (1) clean audio; (2) noisy-test, where the model was trained on clean audio but evaluated on noisy audio; and (3) noise augmentation, where noisy audio was used in both training and inference. All other settings were kept unchanged.

As shown in Table 7, BEATSCORE shows a clear but moderate performance drop when evaluated on noisy audio, confirming that degraded audio quality can affect beat-synchronous segmentation and final score prediction. Nevertheless, the model remains reasonably robust under the noisy-test setting. Moreover, applying noise augmentation during training consistently improves the results over noisy-test, suggesting that the proposed framework can better adapt to realistic noisy conditions when exposed to such perturbations during training.

### 4.6. Analysis of Key Design Choices

Table 8 analyzes several key design choices that control the supervision granularity and the sparsity of grading.

**Hard Negatives for Near-Offset Discrimination:** Using only in-batch negatives provides limited pressure to distinguish aligned pairs from temporally adjacent candidates, because most random negatives are already easy. Adding hard negatives at ±1 beat improves performance, and extending the pool to ±1,±2 typically yields additional gains. This pattern matches the empirical nature of miscoordination in real routines, where visible “off-beat” errors often correspond to half-beat or one-beat deviations rather than large shifts.

**Event Selection and Window Size:** For event selection, a moderate top-*N* works best in practice. Too few events may miss decisive segments, while too many can introduce low-saliency windows that dilute the learning signal and reduce the benefit of sparse grading. A similar trade-off appears for event window size. Small windows can truncate the temporal context needed to judge transitions, whereas overly large windows reintroduce the averaging effect that event-centric grading is designed to avoid. We therefore used an intermediate *N* and *L* as defaults, and the ablation verified that BEATSCORE is not overly sensitive within a reasonable range.

**Pooling Strategy:** MIL pooling consistently improves over mean pooling, indicating that the model benefits from learning instance weights $\pi_{i}$ that emphasize decisive events and downweight irrelevant segments. This is particularly important when the routine contains long stretches of low-difficulty or repetitive motion, where uniform aggregation can blur the evidence that separates close scores.

**Fusion Strategies:** We further compared different fusion strategies for combining global and event-centric grading signals. The results show that both single-branch variants underperform the fusion-based approaches, while the adaptive gate achieves the best overall performance, indicating that input-dependent fusion better balances routine-level and event-level evidence.

### 4.7. Sensitivity Analysis of Key Hyperparameters in BEATSCORE

As shown in Table 9, BEATSCORE is moderately sensitive to both λ and τ, but it remains stable within a reasonable range. When λ is too small, the alignment supervision becomes insufficient; when it is too large, the score regression objective is overly constrained. Similarly, extremely low or high values of τ lead to slightly worse performance, while a moderate temperature yields the best overall results. These observations suggest that the proposed framework does not rely on overly delicate hyperparameter tuning.

### 4.8. Event Evidence Visualization

**Figure 3:**
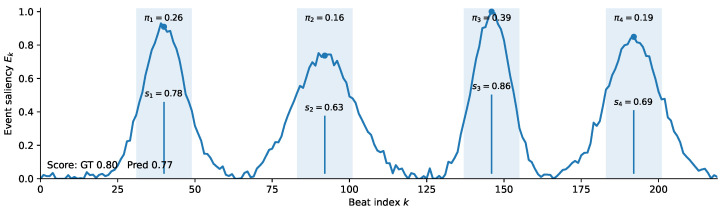
Visualization of event-centric evidence in BEATSCORE on a representative sample. The curve shows event saliency over beats; shaded regions are selected event windows; labels indicate event scores $s_{i}$ and MIL weights $\pi_{i}$.

To better understand *why* BEATSCORE improves long-term assessment, we visualized the event-centric evidence used to form the final prediction in Figure 3. Given a beat-synchronous sequence, the event proposer produces a scalar saliency curve $E_{k}$ over beats *k* (higher means more likely to be a decisive moment). We then select the top-*N* peaks as event centers ${\left\{c_{i}\right\}}_{i=1}^{N}$. For each center $c_{i}$, we extract a local window of radius *L* on the beat axis, i.e., beats in $\left[c_{i}-L,\;c_{i}+L\right]$ (shown as shaded regions), and encode the corresponding audio–motion tokens into an event representation. An event-level regression head outputs an event score $s_{i}$ (annotated near each peak), which captures the local quality and coordination evidence inside that window. Finally, the MIL aggregator assigns a non-negative weight $\pi_{i}$ to each event (annotated above each window) and produces the routine-level prediction as a weighted aggregation of event scores. (We omit the explicit formula in the figure for compactness; the aggregation is implemented as a normalized weighting over $\left\{s_{i}\right\}$.)

Figure 3 shows that the prediction is dominated by a small subset of high-saliency windows: the selected peaks align with local maxima of $E_{k}$, and the learned weights $\pi_{i}$ concentrate on a few events with strong evidence rather than spreading uniformly over the entire routine. This behavior is consistent with the event-driven nature of judging, where a handful of decisive transitions or landings can disproportionately affect the final score. Compared with dense temporal averaging, BEATSCORE provides interpretable attribution by indicating *which* windows contribute most to the predicted score, and this qualitative pattern complements the quantitative gains from event-centric grading and MIL pooling in Table 8.

### 4.9. Controlled Perturbations for Synchrony Verification

**Figure 4:**
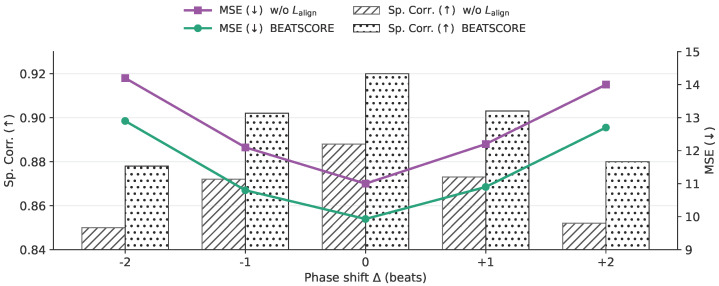
Controlled phase shift on the audio stream. Bars show Avg Spearman correlation (higher is better) and lines show Avg MSE (lower is better). We shift audio by Δ beats while keeping the video unchanged, and we compare BEATSCORE with w/o $L_{\mathrm{align}}$.

To verify that the gain of BEATSCORE indeed comes from learning action–music synchrony, we conducted controlled perturbations on the *audio stream only* while keeping the video unchanged, and then evaluated with the original ground-truth scores. In Figure 4, we apply a global phase shift by Δ beats, where Δ∈{−2,−1,0,+1,+2}, and report both Avg Spearman correlation (bars, higher is better) and Avg MSE (lines, lower is better). Performance peaks at Δ=0 and degrades as |Δ| increases, indicating that the learned predictor is sensitive to rhythmic misalignment instead of being invariant to it. More importantly, BEATSCORE consistently outperforms the variant without beat-level alignment loss ($L_{\mathrm{align}}$) at all shifts, and the gap is most visible around small offsets (e.g., ±1 beat), which matches our design where $L_{\mathrm{align}}$ explicitly contrasts the aligned pair $\left(m_{k},a_{k}\right)$ against near-offset candidates $\left(m_{k},a_{k+\delta }\right)$ for small δ.

**Figure 5:**
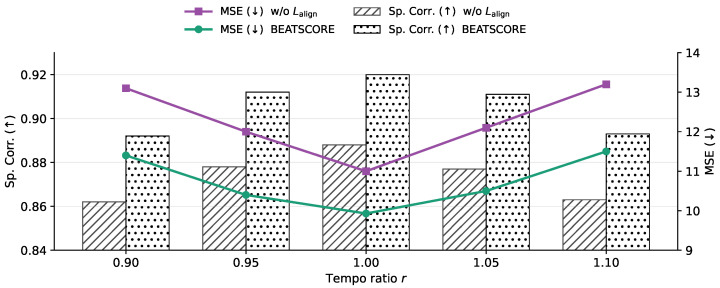
Controlled tempo scaling on the audio stream. We time-stretch audio with ratio *r* while keeping the video unchanged. BEATSCORE consistently achieves higher Avg SRCC and lower Avg MSE than w/o $L_{\mathrm{align}}$.

We further tested tempo perturbations by time-stretching the audio with ratio r∈{0.90,0.95,1.00,1.05,1.10} (Figure 5). Compared to phase shifts, mild tempo changes (e.g., ±5%) lead to smaller accuracy drops, while larger scaling (e.g., ±10%) causes a clearer degradation, consistent with the intuition that global tempo drift perturbs synchrony more gradually than a discrete beat-level phase mismatch. Across all ratios, BEATSCORE maintains higher Avg SRCC and lower Avg MSE than w/o $L_{\mathrm{align}}$, suggesting that explicit beat-level discrimination provides a better inductive bias for long-term scoring under weak audio–motion correlation.

### 4.10. Sensitivity to Beat-Axis Quality

**Figure 6:**
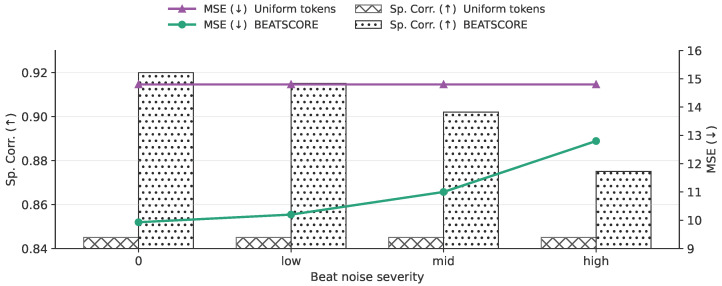
Sensitivity to beat-axis quality. We corrupted detected beat timestamps before beat-synchronous tokenization with jitter/drop/insert noise (shown by severity). The uniform-token baseline does not use beat information.

BEATSCORE relies on an off-the-shelf beat tracker to define the beat axis for beat-synchronous tokenization. We therefore analyze sensitivity to beat quality by injecting controlled noise into the detected beat timestamps before tokenization (Figure 6). We consider three noise types: (i) *jitter*, which adds i.i.d. temporal noise $\epsilon ∼N(0,\sigma^{2})$ to each beat time; (ii) *drop*, which removes each beat independently with probability *p*; and (iii) *insert*, which adds spurious beats uniformly between neighboring beats with probability *p*. We map these perturbations to a unified severity level (from 0 to high) for compact visualization. As severity increases, performance decreases smoothly, and BEATSCORE remains above a uniform-token baseline that does not use beat information, indicating that the model benefits from a beat axis without collapsing under moderate beat imperfections. This sensitivity analysis complements our main ablations by directly addressing the practical question of beat-tracking errors in real routines.

**Figure 7:**
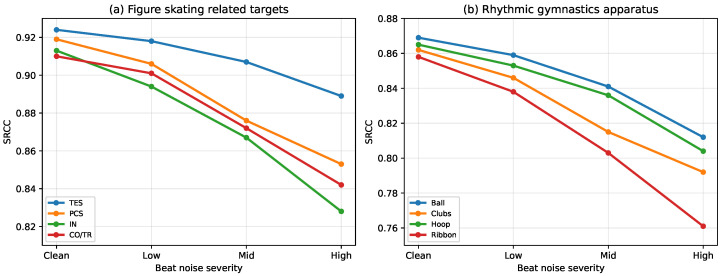
Sensitivity to beat-axis quality. We corrupted detected beat timestamps before beat-synchronous tokenization with jitter/drop/insert noise (shown by severity). The uniform-token baseline does not use beat information.

Figure 7 extends Figure 6 by reporting beat-noise sensitivity separately for different scoring dimensions and sub-events. We can observe that the performance drop is not uniform across targets. In figure skating, coordination-sensitive components generally show a larger degradation than technical scores as beat noise increases. In rhythmic gymnastics, different apparatus categories also exhibit distinct sensitivity patterns. These results suggest that beat-aware alignment is particularly beneficial for evaluation targets that depend more strongly on rhythm consistency and action–music coordination.

## 5. Conclusions

We have presented BEATSCORE, a beat-guided audio–visual framework for long-term sports assessment. BEATSCORE makes action–music coordination explicitly learnable by aligning audio and motion on a beat axis and training near-offset contrastive discrimination, and it matches routine-level scoring to sparse decisive evidence via event-centric grading and MIL aggregation. Across FS1000, Fis-V, and RG, BEATSCORE consistently improves assessment accuracy under standard protocols, and ablations verify that beat-level alignment and event-centric aggregation contribute complementary gains.

**Limitations and Future Directions:** First, the framework relies on a beat tracker to define the beat axis. While we evaluated robustness under controlled beat perturbations, performance can still degrade when the audio is noisy, heavily edited, or weakly metrical, where beat boundaries are less stable. Second, beat-synchronous tokens impose a fixed temporal granularity. Some scoring evidence is off-beat (e.g., gradual drift, sustained control, or transitions that span multiple beats) and may not be fully captured by a per-beat representation. Third, our event proposal is driven by motion-change saliency. This is effective for sharp technical events, but it can underweight errors that occur during low-motion segments or overemphasize visually salient movements that are not decisive for judging.

## Figures and Tables

**Table 1:** **FS1000** comparisons with prior methods; † indicates using audio information. **Setting** summarizes the input modality and backbone: V/V+A denote visual-only and audio–visual inputs, respectively; TF is TimeSformer; AST is Audio Spectrogram Transformer.

Methods	Year	Features	Spearman Correlation (↑)								Mean Square Error (↓)							
			TES	PCS	SS	TR	PE	CO	IN	Avg.	TES	PCS	SS	TR	PE	CO	IN	Avg.
C3D-LSTM [44]	2017	V (C3D [45])	0.78	0.53	0.50	0.52	0.52	0.57	0.47	0.57	308.30	25.85	0.92	0.99	1.21	0.97	1.01	48.61
MSCADC [17]	2019	V (TF [6])	0.77	0.70	0.69	0.69	0.71	0.68	0.71	0.71	148.02	15.47	0.51	0.57	0.78	0.55	0.60	23.79
MS-LSTM [1]	2019	V (TF [6])	0.86	0.80	0.77	0.78	0.76	0.79	0.78	0.79	94.55	11.03	0.45	0.49	0.76	0.43	0.47	15.45
CoRe [34]	2021	V (TF [6])	0.88	0.84	0.81	0.83	0.81	0.83	0.80	0.83	103.50	9.85	0.41	0.37	0.81	0.38	0.41	16.53
GDLT [22]	2022	V (TF [6])	0.88	0.86	0.84	0.86	0.83	0.85	0.84	0.85	82.73	10.32	0.35	0.37	0.67	0.38	0.42	13.60
TPT [13]	2022	V (TF [6])	0.88	0.83	0.82	0.83	0.81	0.82	0.81	0.83	80.00	8.88	0.34	0.37	0.63	0.34	0.39	12.99
T2CR [21]	2024	V (TF [6])	0.86	0.79	0.83	0.84	0.82	0.84	0.80	0.83	107.59	15.26	0.61	0.48	0.69	0.57	0.42	17.95
CoFInAl [46]	2024	V (TF [6])	0.84	0.83	0.84	0.84	0.81	0.83	0.82	0.83	81.65	16.05	0.56	0.63	0.71	0.41	0.54	14.36
QTD [14]	2024	V (TF [6])	0.88	0.85	0.85	0.86	0.83	0.85	0.84	0.85	137.09	17.48	0.51	0.73	0.80	0.91	0.98	22.64
M-BERT (Late) [27] †	2020	V+A (TF [6] + AST [47])	0.79	0.75	0.80	0.81	0.80	0.80	0.76	0.79	131.28	15.28	0.44	0.43	0.67	0.47	0.55	21.30
MLP-Mixer [2] †	2023	V+A (TF [6] + AST [47])	0.88	0.82	0.80	0.81	0.80	0.81	0.81	0.82	81.24	9.47	0.35	0.35	0.62	0.37	0.39	13.26
SGN [3] †	2024	V+A (TF [6] + AST [47])	0.89	0.85	0.84	0.85	0.82	0.85	0.83	0.85	79.08	8.40	0.31	0.32	0.61	0.33	0.37	12.77
PAMFN [9] †	2024	V+A (TF [6] + AST [47] + I3D [5])	0.90	0.89	0.86	0.87	0.86	0.87	0.85	0.87	104.89	10.05	0.39	0.52	0.78	0.40	0.56	16.80
MLAVL [12] †	2025	V+A (TF [6] + AST [47] + CLIP [38])	0.92	0.89	0.90	0.90	0.88	0.89	0.88	0.90	64.89	6.39	0.23	0.24	0.50	0.25	0.26	10.39
BEATSCORE† (Ours)	-	V+A (TF [6] + AST [47])	**0.93**	**0.90**	**0.92**	**0.91**	**0.90**	**0.91**	**0.92**	**0.92**	**59.14**	**5.87**	**0.22**	**0.21**	**0.46**	**0.21**	**0.22**	**9.93**

**Table 2:** Comparison on Fis-V.

			Sp. Corr. (↑)						MSE (↓)					
Methods	# Params	# FLOPs	Val			Test			Val			Test		
			TES	PCS	Avg.	TES	PCS	Avg.	TES	PCS	Avg.	TES	PCS	Avg.
C3D-LSTM [44]			0.279	0.498	0.399	0.290	0.510	0.406	29.21	21.87	30.67	39.25	21.97	30.61
MSCADC [17]			0.477	0.602	0.535	0.500	0.610	0.557	25.84	11.72	18.59	25.93	11.94	18.94
MS-LSTM [1]			0.661	0.790	0.742	0.650	0.780	0.721	19.88	8.29	14.05	19.91	8.35	14.13
M-BERT (Late) [27]	4.00	1.272	0.529	0.713	0.633	0.530	0.720	0.634	27.77	12.40	20.11	27.73	12.38	20.06
GDLT [22]	3.20	0.268	0.690	0.837	0.769	0.685	0.820	0.761	20.32	8.74	14.82	20.99	8.75	14.87
CoRe [34]	2.51	0.010	0.654	0.812	0.726	0.660	0.820	0.751	23.51	9.29	16.40	23.50	9.25	16.38
TPT [13]	11.82	2.229	0.552	0.462	0.648	0.570	0.760	0.676	27.53	11.26	19.41	27.50	11.25	19.38
MLP-Mixer [2]	14.32	49.900	0.674	0.831	0.754	0.680	0.820	0.759	19.55	7.89	13.62	19.57	7.96	13.77
SGN [3]			0.714	0.837	0.791	0.700	0.830	0.773	19.03	7.96	13.50	19.05	7.96	13.51
PAMFN [9]	18.06	2.562	0.766	0.877	0.825	0.754	0.872	0.822	22.49	8.20	15.31	22.50	8.16	15.33
CoFInAl [46]	5.24	0.509	0.713	0.839	0.771	0.716	0.843	0.788	20.74	7.85	14.28	20.76	7.91	14.34
QTD [14]	5.51	0.396	0.706	0.856	0.790	0.717	0.858	0.798	26.99	10.91	18.90	26.97	10.89	18.93
MLAVL [12]	3.82	0.778	0.769	0.862	0.820	0.766	0.863	0.823	19.41	7.15	13.29	19.44	7.17	13.31
BEATSCORE (Ours)	**3.47**	**0.760**	**0.797**	**0.889**	**0.860**	**0.795**	**0.883**	**0.851**	**18.31**	**6.87**	**13.05**	**18.32**	**6.94**	**13.06**

**Table 3:** Comparison on Rhythmic Gymnastics (RG). Avg. denotes the arithmetic mean over the four apparatus categories.

Methods	Year	Features	Spearman Correlation (↑)					Mean Square Error (↓)				
			Ball	Clubs	Hoop	Ribbon	Avg.	Ball	Clubs	Hoop	Ribbon	Avg.
C3D+SVR [44]	2017	C3D [45]	0.357	0.551	0.495	0.516	0.483	–	–	–	–	–
MS-LSTM [1]	2019	I3D [5]	0.515	0.621	0.540	0.522	0.551	10.55	6.94	5.85	12.56	8.97
MS-LSTM [1]	2019	VST [7]	0.621	0.661	0.670	0.695	0.663	7.52	6.04	6.16	5.78	6.37
ACTION-NET [4]	2020	I3D [5] + ResNet [48]	0.528	0.652	0.708	0.578	0.623	9.09	6.40	5.93	10.23	7.91
ACTION-NET [4]	2020	VST [7] + ResNet [48]	0.684	0.737	0.733	0.754	0.728	9.55	6.36	5.56	8.15	7.41
GDLT [22]	2022	VST [7]	0.746	0.802	0.765	0.741	0.765	5.90	4.34	5.70	6.16	5.53
PAMFN [9]	2024	VST [7] + AST [47] + I3D [5]	0.757	0.825	0.836	0.846	0.819	6.24	7.45	5.21	7.67	6.64
CoFInAl [46]	2024	I3D [5]	0.625	0.719	0.734	0.757	0.712	7.04	6.37	5.81	6.98	6.55
CoFInAl [46]	2024	VST [7]	0.809	0.806	0.804	0.810	0.807	5.07	5.19	6.37	6.30	5.73
QTD [22]	2024	VST [7]	0.823	0.852	0.837	0.857	0.842	7.94	5.66	7.95	8.87	7.61
MLAVL [12]	2025	VST [7] + AST [47] + CLIP [38]	0.826	0.829	0.871	0.866	0.849	5.57	4.20	4.11	3.99	4.47
BEATSCORE (Ours)	-	VST + AST	**0.841**	**0.855**	**0.887**	**0.872**	**0.864**	**4.33**	**4.05**	**4.09**	**3.68**	**4.04**

**Table 4:** Impact of different beat trackers on BEATSCORE; ^†^ denotes the default beat tracker used in our main experiments.

Beat Tracker	FS1000 (Avg.)		RG (Avg.)	
	SRCC ↑	MSE ↓	SRCC ↑	MSE ↓
librosa ^†^	**0.92**	**9.93**	**0.86**	**4.17**
Essentia	0.91	10.21	0.85	4.36
aubio	0.90	10.58	0.84	4.62

**Table 5:** Effects of different event proposal strategies; ^†^ denotes the default event proposal strategy used in our main experiments.

Event Proposal	FS1000 (Avg.)		RG (Avg.)	
	SRCC ↑	MSE ↓	SRCC ↑	MSE ↓
Motion-only ^†^	**0.92**	9.93	**0.86**	**4.17**
Audio-only	0.62	30.67	0.59	8.26
Motion + audio	0.90	**9.95**	**0.85**	**4.17**

**Table 6:** Effects of decomposing action quality and action–music coordination signals in event-centric grading; ^†^ denotes the full BEATSCORE model used in our main experiments.

Scoring Signal	FS1000 (Avg.)		RG (Avg.)	
	SRCC ↑	MSE ↓	SRCC ↑	MSE ↓
Quality-only	0.89	10.21	0.85	4.37
Coordination-only	0.81	13.05	0.76	5.65
Quality + coordination ^†^	**0.92**	**9.93**	**0.86**	**4.17**

**Table 7:** Robustness of BEATSCORE under noisy audio conditions; ^†^ denotes the default setting used in our main experiments.

Audio Setting	FS1000 (Avg.)		RG (Avg.)	
	SRCC ↑	MSE ↓	SRCC ↑	MSE ↓
Clean audio ^†^	**0.92**	**9.93**	**0.86**	**4.17**
Noisy-test	0.84	14.55	0.71	6.72
Noise augmentation	0.88	14.07	0.84	5.50

**Table 8:** Effect of key design choices in BEATSCORE, including beat-level alignment, event-centric grading, pooling, and fusion strategies.

Variant	FS1000 (Avg.)		RG (Avg.)	
	SRCC ↑	MSE ↓	SRCC ↑	MSE ↓
*Beat-level contrastive negatives*				
In-batch negatives only	0.89	11.41	0.84	5.07
Hard negatives (±1)	0.90	11.37	0.85	4.45
Hard negatives (±1,±2)	**0.92**	**9.93**	**0.86**	**4.17**
*Event selection*				
Top-*N* peaks (N=2)	0.90	10.65	0.84	5.14
Top-*N* peaks (N=4)	**0.92**	**9.93**	**0.86**	**4.17**
Top-*N* peaks (N=8)	0.84	14.00	0.77	7.96
*Event window size*				
Window radius L=2 (size 2L+1)	0.89	11.42	0.85	4.60
Window radius L=3 (size 2L+1)	**0.92**	**9.93**	**0.86**	**4.17**
Window radius L=4 (size 2L+1)	0.87	11.79	0.84	6.01
*Pooling*				
Mean pooling	0.87	12.04	0.82	6.25
MIL pooling (ours)	**0.92**	**9.93**	**0.86**	**4.17**
*Fusion*				
Global-only	0.58	48.72	0.43	28.15
Event-only	0.83	15.20	0.76	14.02
Fixed average	0.88	15.64	0.80	17.74
Adaptive gate (ours)	**0.92**	**9.93**	**0.86**	**4.17**

**Table 9:** Sensitivity analysis of key hyperparameters in BEATSCORE. We varied the alignment weight λ and the contrastive temperature τ while keeping all other settings unchanged.

Hyperparameter	FS1000 (Avg.)		RG (Avg.)	
	SRCC ↑	MSE ↓	SRCC ↑	MSE ↓
*Alignment weight λ*				
λ=0.05	0.83	12.93	0.74	6.31
λ=0.10	**0.92**	**9.93**	**0.86**	**4.17**
λ=0.20	0.91	10.22	0.84	4.55
*Contrastive temperature τ*				
τ=0.05	0.90	10.41	0.85	4.68
τ=0.10	**0.92**	**9.93**	**0.86**	**4.17**
τ=0.20	0.87	16.75	0.82	12.55

## Data Availability

No new data were created or analyzed in this study.

## Abbreviations

The following abbreviations are used in this manuscript:

BEATSCORE	Beat-Synchronous Contrastive Alignment and Event-Centric Grading for Long-Term Sports Assessment
BEATTOK	Beat-Synchronous Tokenization (Beat-Axis Tokenization)
AQA	Action Quality Assessment
InfoNCE	(Information) Noise-Contrastive Estimation
NMS	Non-Maximum Suppression
MIL	Multiple Instance Learning
MLP	Multi-Layer Perceptron
SRCC	Spearman Rank Correlation Coefficient
MSE	Mean Squared Error
FS1000	Figure Skating 1000 Benchmark/Dataset
Fis-V	Figure Skating video Benchmark/Dataset
RG	Rhythmic Gymnastics Benchmark/Dataset
TES	Technical Element Score
PCS	Program Component Score
SS	Skating Skills
TR	Transitions
PE	Performance
CO	Composition
IN	Interpretation of Music
TF	TimeSformer
VST	Video Swin Transformer
AST	Audio Spectrogram Transformer
I3D	Inflated 3D ConvNet
C3D	3D Convolutional Network
CLIP	Contrastive Language–Image Pre-Training
fps	Frames per Second

## References

[1] Xu C., Fu Y., Zhang B., Chen Z., Jiang Y.G., Xue X. (2019). Learning to score figure skating sport videos. IEEE Trans. Circuits Syst. Video Technol..

[2] Xia J., Zhuge M., Geng T., Fan S., Wei Y., He Z., Zheng F. (2023). Skating-mixer: Long-term sport audio-visual modeling with mlps. Proc. AAAI Conf. Artif. Intell..

[3] Du Z., He D., Wang X., Wang Q. (2023). Learning semantics-guided representations for scoring figure skating. IEEE Trans. Multimed..

[4] Zeng L.A., Hong F.T., Zheng W.S., Yu Q.Z., Zeng W., Wang Y.W., Lai J.H. Hybrid dynamic-static context-aware attention network for action assessment in long videos. Proceedings of the 28th ACM international Conference on Multimedia.

[5] Carreira J., Zisserman A. Quo vadis, action recognition? A new model and the kinetics dataset. Proceedings of the IEEE Conference on Computer Vision and Pattern Recognition.

[6] Bertasius G., Wang H., Torresani L. Is space-time attention all you need for video understanding?. Proceedings of the 38th International Conference on Machine Learning.

[7] Liu Z., Ning J., Cao Y., Wei Y., Zhang Z., Lin S., Hu H. Video swin transformer. Proceedings of the IEEE/CVF Conference on Computer Vision and Pattern Recognition.

[8] Liu X., Meng S., Li Q., Qi L., Xu X., Dou W., Zhang X. Smef: Social-aware multi-dimensional edge features-based graph representation learning for recommendation. Proceedings of the 32nd ACM International Conference on Information and Knowledge Management.

[9] Zeng L.A., Zheng W.S. (2024). Multimodal action quality assessment. IEEE Trans. Image Process..

[10] Li X., Bhattacharjya A., Li Q., Zhou M., Wisniewski R., Bazydło G. (2026). A novel method for vulnerability detection based on fusion and hyperbolic neural network graphs. IEEE Trans. Softw. Eng..

[11] Liu X., Meng S., Li Q., Liu Q., He Q., Ramesh D., Qi L. (2023). FDGNN: Feature-aware disentangled graph neural network for recommendation. IEEE Trans. Comput. Soc. Syst..

[12] Xu H., Ke X., Wu H., Xu R., Li Y., Guo W. Language-Guided Audio-Visual Learning for Long-Term Sports Assessment. Proceedings of the Computer Vision and Pattern Recognition Conference.

[13] Bai Y., Zhou D., Zhang S., Wang J., Ding E., Guan Y., Long Y., Wang J. (2022). Action quality assessment with temporal parsing transformer. Proceedings of the European Conference on Computer Vision.

[14] Dong X., Liu X., Li W., Adeyemi-Ejeye A., Gilbert A. (2024). Interpretable long-term action quality assessment. arXiv.

[15] Zhang S., Dai W., Wang S., Shen X., Lu J., Zhou J., Tang Y. Logo: A long-form video dataset for group action quality assessment. Proceedings of the IEEE/CVF Conference on Computer Vision and Pattern Recognition.

[16] Pirsiavash H., Vondrick C., Torralba A. (2014). Assessing the quality of actions. Proceedings of the European Conference on Computer Vision.

[17] Parmar P., Morris B. (2019). Action quality assessment across multiple actions. Proceedings of the 2019 IEEE Winter Conference on Applications of Computer Vision (WACV).

[18] Xu J., Rao Y., Yu X., Chen G., Zhou J., Lu J. Finediving: A fine-grained dataset for procedure-aware action quality assessment. Proceedings of the IEEE/CVF Conference on Computer Vision and Pattern Recognition.

[19] Xu J., Yin S., Zhao G., Wang Z., Peng Y. Fineparser: A fine-grained spatio-temporal action parser for human-centric action quality assessment. Proceedings of the IEEE/CVF Conference on Computer Vision and Pattern Recognition.

[20] Xarles A., Escalera S., Moeslund T.B., Clapés A. Astra: An action spotting transformer for soccer videos. Proceedings of the 6th International Workshop on Multimedia Content Analysis in Sports.

[21] Ke X., Xu H., Lin X., Guo W. (2024). Two-path target-aware contrastive regression for action quality assessment. Inf. Sci..

[22] Xu A., Zeng L.A., Zheng W.S. Likert scoring with grade decoupling for long-term action assessment. Proceedings of the IEEE/CVF Conference on Computer Vision and Pattern Recognition.

[23] Doughty H., Mayol-Cuevas W., Damen D. The pros and cons: Rank-aware temporal attention for skill determination in long videos. Proceedings of the IEEE/CVF Conference on Computer Vision and Pattern Recognition.

[24] Xu H., Ke X., Li Y., Xu R., Wu H., Lin X., Guo W. (2024). Vision-language action knowledge learning for semantic-aware action quality assessment. Proceedings of the European Conference on Computer Vision.

[25] Xu H., Wu H., Ke X., Li Y., Xu R., Guo W. (2025). Quality-guided vision-language learning for long-term action quality assessment. IEEE Trans. Multimed..

[26] Zhang S., Bai S., Chen G., Chen L., Lu J., Wang J., Tang Y. Narrative action evaluation with prompt-guided multimodal interaction. Proceedings of the IEEE/CVF Conference on Computer Vision and Pattern Recognition.

[27] Lee S., Yu Y., Kim G., Breuel T., Kautz J., Song Y. (2020). Parameter efficient multimodal transformers for video representation learning. arXiv.

[28] Alfasly S., Lu J., Xu C., Zou Y. Learnable irrelevant modality dropout for multimodal action recognition on modality-specific annotated videos. Proceedings of the IEEE/CVF Conference on Computer Vision and Pattern Recognition.

[29] Nagrani A., Yang S., Arnab A., Jansen A., Schmid C., Sun C. (2021). Attention bottlenecks for multimodal fusion. Adv. Neural Inf. Process. Syst..

[30] Chen J., Ho C.M. Mm-vit: Multi-modal video transformer for compressed video action recognition. Proceedings of the IEEE/CVF Winter Conference on Applications of Computer Vision.

[31] Chi L., Tian G., Mu Y., Tian Q. Two-stream video classification with cross-modality attention. Proceedings of the IEEE/CVF International Conference on Computer Vision Workshops.

[32] Lin Y.B., Tseng H.Y., Lee H.Y., Lin Y.Y., Yang M.H. (2021). Exploring cross-video and cross-modality signals for weakly-supervised audio-visual video parsing. Adv. Neural Inf. Process. Syst..

[33] Vaswani A., Shazeer N., Parmar N., Uszkoreit J., Jones L., Gomez A.N., Kaiser Ł., Polosukhin I. (2017). Attention is all you need. Adv. Neural Inf. Process. Syst..

[34] Yu X., Rao Y., Zhao W., Lu J., Zhou J. Group-aware contrastive regression for action quality assessment. Proceedings of the IEEE/CVF International Conference on Computer Vision.

[35] Morgado P., Vasconcelos N., Misra I. Audio-visual instance discrimination with cross-modal agreement. Proceedings of the IEEE/CVF Conference on Computer Vision and Pattern Recognition.

[36] Alwassel H., Mahajan D., Korbar B., Torresani L., Ghanem B., Tran D. (2020). Self-supervised learning by cross-modal audio-video clustering. Adv. Neural Inf. Process. Syst..

[37] Arandjelovic R., Zisserman A. Look, listen and learn. Proceedings of the IEEE International Conference on Computer Vision.

[38] Radford A., Kim J.W., Hallacy C., Ramesh A., Goh G., Agarwal S., Sastry G., Askell A., Mishkin P., Clark J. (2021). Learning transferable visual models from natural language supervision. Proceedings of the International Conference on Machine Learning.

[39] Tan R., Ray A., Burns A., Plummer B.A., Salamon J., Nieto O., Russell B., Saenko K. Language-guided audio-visual source separation via trimodal consistency. Proceedings of the IEEE/CVF Conference on Computer Vision and Pattern Recognition.

[40] Liang S., Huang C., Tian Y., Kumar A., Xu C. Language-guided joint audio-visual editing via one-shot adaptation. Proceedings of the Asian Conference on Computer Vision.

[41] Rasheed H., Khattak M.U., Maaz M., Khan S., Khan F.S. Fine-tuned clip models are efficient video learners. Proceedings of the IEEE/CVF Conference on Computer Vision and Pattern Recognition.

[42] Ju C., Zheng K., Liu J., Zhao P., Zhang Y., Chang J., Tian Q., Wang Y. Distilling vision-language pre-training to collaborate with weakly-supervised temporal action localization. Proceedings of the IEEE/CVF Conference on Computer Vision and Pattern Recognition.

[43] Lim G., Kim H., Kim J., Choi Y. Probabilistic vision-language representation for weakly supervised temporal action localization. Proceedings of the 32nd ACM International Conference on Multimedia.

[44] Parmar P., Tran Morris B. Learning to score olympic events. Proceedings of the IEEE Conference on Computer Vision and Pattern Recognition Workshops.

[45] Tran D., Bourdev L., Fergus R., Torresani L., Paluri M. Learning spatiotemporal features with 3d convolutional networks. Proceedings of the IEEE International Conference on Computer Vision.

[46] Zhou K., Li J., Cai R., Wang L., Zhang X., Liang X. (2024). Cofinal: Enhancing action quality assessment with coarse-to-fine instruction alignment. arXiv.

[47] Gong Y., Chung Y.A., Glass J. (2021). Ast: Audio spectrogram transformer. arXiv.

[48] He K., Zhang X., Ren S., Sun J. Deep residual learning for image recognition. Proceedings of the IEEE Conference on Computer Vision and Pattern Recognition.

